# Probing long-range interactions by extracting free energies from genome-wide chromosome conformation capture data

**DOI:** 10.1186/s12859-015-0584-2

**Published:** 2015-05-23

**Authors:** Saeed Saberi, Pau Farré, Olivier Cuvier, Eldon Emberly

**Affiliations:** 1 Physics Department, Simon Fraser University, 8888 University Drive, Burnaby, V5A 1S6 BC Canada; 2 Laboratoire de Biologie Moléculaire des Eucaryotes (LBME), Toulouse, France

**Keywords:** Chromatin, Hi-C, PCA, ChIP, DNA looping, Insulators

## Abstract

**Background:**

A variety of DNA binding proteins are involved in regulating and shaping the packing of chromatin. They aid the formation of loops in the DNA that function to isolate different structural domains. A recent experimental technique, Hi-C, provides a method for determining the frequency of such looping between all distant parts of the genome. Given that the binding locations of many chromatin associated proteins have also been measured, it has been possible to make estimates for their influence on the long-range interactions as measured by Hi-C. However, a challenge in this analysis is the predominance of non-specific contacts that mask out the specific interactions of interest.

**Results:**

We show that transforming the Hi-C contact frequencies into free energies gives a natural method for separating out the distance dependent non-specific interactions. In particular we apply Principal Component Analysis (PCA) to the transformed free energy matrix to identify the dominant modes of interaction. PCA identifies systematic effects as well as high frequency spatial noise in the Hi-C data which can be filtered out. Thus it can be used as a data driven approach for normalizing Hi-C data. We assess this PCA based normalization approach, along with several other normalization schemes, by fitting the transformed Hi-C data using a pairwise interaction model that takes as input the known locations of bound chromatin factors. The result of fitting is a set of predictions for the coupling energies between the various chromatin factors and their effect on the energetics of looping. We show that the quality of the fit can be used as a means to determine how much PCA filtering should be applied to the Hi-C data.

**Conclusions:**

We find that the different normalizations of the Hi-C data vary in the quality of fit to the pairwise interaction model. PCA filtering can improve the fit, and the predicted coupling energies lead to biologically meaningful insights for how various chromatin bound factors influence the stability of DNA loops in chromatin.

**Electronic supplementary material:**

The online version of this article (doi:10.1186/s12859-015-0584-2) contains supplementary material, which is available to authorized users.

## Background

Eukaryotes organize their DNA on a range of length scales into a packaged structure known as chromatin. At the smallest length scale, DNA is wrapped around histones to form nucleosomes that aid the condensing of the DNA. Histones can be chemically modified, that depending on the type of modification, can mark the chromatin as either being in a silent, heterochromatic state or active, euchromatic state. These histone modifications are passed down from one cell to the next, thus forming one part of a cell’s epigenetic regulatory machine [1,2]. On longer length scales chromosomes fold into topological domains in the space of the nucleus. Such organization may contribute to separate heterochromatin and euchromatin via the formation of sequestering loops (ranging from 1 to 500 kbp in length) within each type of domain [2]. The likelihood of long-range contacts between distant loci involves specific factors/proteins participating in chromatin organization, thereby mediating the contact frequency of specific DNA loops [3,4]. Some of these proteins condense the DNA making heterochromatin regions [5] while others are associated with euchromatin [2,3,6-12]. It remains unclear to what extent their impact on looping is a result of being in a particular epigenetic background, as euchromatin and heterochromatin may influence long range interactions as well.

The recent development of the High-throughput Chromosome capture method (Hi-C) [13-18] has provided a valuable tool to study the 3D organization of chromosomes on a genome-wide level. This method measures the frequency of contact between any two segments along the genome. Using this data, a variety of methods have aimed to predict the underlying 3D structure of the chromosomes [15,19-22]. On large scales, DNA confinement plays a role in structuring the chromatin, and modeling has shown that such effects can lead to inheritable territories [23]. Nevertheless, many studies have also shown that the overall organization found in the Hi-C data correlates with the underlying domain structure of the chromatin and the corresponding bound proteins in those domains [13,14,20] as identified by Chromatin immunoprecipitation (ChIP-chip or ChIP-seq). It has thus been possible to intersect these two data sets to infer the influence of chromatin associated proteins in long-range interactions. As such, it has been shown that the insulator protein CTCF facilitates looping between distant sites provided the additional presence of Cohesin and/or mediator complexes [24]. CTCF has been found to be enriched at boundaries between heterochromatic and euchromatic domains and sometimes aids the regulation of enhancer-promoter interactions [3,24]. In Drosophila, a number of additional insulator proteins that bind insulator sequences have been identified: BEAF32, dCTCF, GAF, Zw5 and Su(Hw). They have been found to interact with each other thereby stabilizing long-range interactions among distant insulator sites [25]. Such looping involves further insulator protein cofactors such as CP190, Chromator or Cohesin [14,24,25]. These insulator proteins form a network of interactions that may contribute to structure and isolate active domains from inactive chromatin within the Drosophila genome [24].

The assembly of chromatin into silent domains has a similar network of interactions that are confined within such domains [14] or that involve long-range interactions between distant silent domains [26]. Important contributors to these interactions within heterochromatin are the PolyComb-Group (PCG) proteins that play key roles in the spreading of the silent state upon binding of PCG and co-factors to specific DNA sequences called Polycomb Response Elements (PREs). Hence, analogous to how insulators aid the structuring of euchromatin domains, PCG proteins have their own associated set of interactions that aid the formation of heterochromatin.

Here we aim to quantify the effective energetics of interaction between different chromatin regulators from the measured Hi-C contact frequencies. For this purpose it is crucial to disentangle the effect of different contributing factors within the Hi-C data itself. The largest contributing factor to the observed frequency of contacts in Hi-C data is the distance dependent likelihood of contact between loci due solely to the polymer nature of the DNA. This distance dependent likelihood acts as a background that helps to hide the specific contacts that exist between chromatin regulatory factors. The distance dependent scaling of this background contact frequency has been shown to be consistent with a confined polymer model [23,27-29]. Other contributing factors to Hi-C data are systematic biases introduced as a result of the nature of the experimental protocol. For example, it is known that the Hi-C procedure generates biases due to the sequence and length of contacting DNA segments [14]. Thus, depending on various DNA features, some loci may be observed more frequently than expected. Several normalization methods have been proposed to correct for these biases [14,30,31] (for a Review see [32]). However, which normalization method provides the most significant information between the specific contact frequencies and the underlying bound factors has not been surveyed in detail before.

In this paper we introduce a method for transforming the measured Hi-C contact frequencies into free energies. The method is based on an equilibrium statistical mechanics approach, where we assume that the frequency of contact between two genomic locations is related to the free energy of forming that particular contact state. Due to the additive nature of free energy, Principal Component Analysis (PCA) provides a convenient tool for then decomposing these free energies into a set of independent modes of interaction. PCA identifies a length dependent background looping energy, systematic biases, and then a series of modes of increasing spatial frequency with which to express the data. We can reconstruct the transformed Hi-C data using the PCs, leaving those out that are due to systematic biases as well as those which are high frequency noise. Our approach is a data driven method for normalizing Hi-C data.

We assess our normalization scheme, as well as two other methods by fitting the free energy data to an interaction model involving the locations of known DNA bound chromatin factors. We model the energy of interaction between two loci as a linear superposition of pairwise interactions between all the bound chromatin factors at those two locations. Given that the energy of interaction and the bound occupancies for the various factors are measured, the model can be fit to predict the couplings between factors. Our fitted couplings show a complex interplay of interactions between the chromatin factors, capturing many known biological relationships. We use the quality of fit of the model as a criterion to determine how many PCs should be filtered. Interestingly we find that other normalization schemes that correct for various biases are less well fit by this pairwise model than our PCA based normalization scheme.

## Methods

### Genomic datasets

We have used the Hi-C dataset for Drosophila Melanogaster reported in Sexton et al. [14]. This data consists of a list of genomic locations for pairs of sequences that were found to be in contact, and the number of times each sequence pair was sequenced. We have also downloaded genome wide binding profiles and enriched binding regions from modencode.org for the following chromatin factors (insulators: BEAF, CP190, dCTCF, GAF, ZW5, epigenetic marks: H3K27Me3, H3K4me3; dosage compensation complex: MOF; PolyComb-Group proteins: Pc, Pho, PCL2; Cohesin: SMC3; Other factors: Nurf, Chromator, PolII) [33]. All coordinates are with respect to Release 5 of the Drosophila Melanogaster genome.

### Free energy matrix

Hi-C measures the number of times two genomic locations come into contact. From this data, a contact matrix can be built at a given level of resolution. The genome is partitioned into non-overlapping bins of fixed size (i.e. 10 kb, 20 kb etc) and the contact matrix element, *C*_*i*,*j*_ is the number of times sequences in bin *i* were found to be in contact with sequences in bin *j*. Using the data from Sexton et al. we have constructed a contact matrix at 10 kb resolution for the *Drosophila Melanogaster* genome. (Following the approach of Sexton et al., for each sequence pair we only count the contribution to a particular *C*_*i*,*j*_ element once, rather than the number of times it was sequenced. This is argued to remove some of the sequence dependent bias in the Hi-C protocol).

Assuming the Hi-C measurements represent an equilibrium distribution, we can associate the contact frequency between bins *i* and *j* with a free energy, *F*_*i*,*j*_, via *C*_*i*,*j*_∝ exp(−*F*_*i*,*j*_/*k*_*B*_ *T*). Thus we can transform the above contact matrix into a matrix of free energies defined by,

(1) $$\begin{array}{@{}rcl@{}}  \frac{F_{i,j}}{k_{B} T} &= -\log(C_{i,j}) + F_{0}. \end{array} $$

We set *k*_*B*_ *T*=1 for the sake of simplicity in the rest of this study, and set *F*_0_=0 as it just defines a reference energy. This free energy contains both an energetic (enthalpic) contribution, arising from specific interactions between DNA bound factors, and an entropic contribution, that is due to the assortment of conformations that the polymer of DNA can adopt. (We have added a pseudocount of 1 to all *C*_*i*,*j*_ to fill in locations *i*,*j* where the contact matrix was zero. Other methods to fill in missing values, such as interpolating between *F*_*i*,*j*_, yield similar results).

### Normalizing the Hi-C contact matrix

The contact matrix created from the *raw* Hi-C data is not corrected for any potential systematic biases (aside from counting each sequence pair only once). Before applying the free energy transformation, Eq. 1, we also have used two separate normalization procedures that correct for biases in the data. The first method, ICE (see [30] for details), normalizes the contact matrix so that each bin has the same number of interactions as any other. The second method that we use was introduced in Sexton et al. [14] and uses a probabilistic model to correct for various systematic biases. This method does not normalize all the bins to have the same number of interactions genome-wide.

### Free energy decomposition: principal component analysis based normalization

The free energy, *F*_*i*,*j*_ between bin *i* and *j*, can be decomposed into two terms,

(2) $$\begin{array}{@{}rcl@{}}  F_{i,j}&=\bar{F}_{j-i}+\delta F_{i,j}, \end{array} $$

where $\bar {F}_{j-i}$ is the average free energy at a fixed genomic distance, *j*−*i*, and is found by averaging over all such distances genome-wide, and *δ* *F*_*i*,*j*_ is the free energy difference from this average that depends on the two interacting bins. The genome-wide average free energy, is computed via $\bar {F}_{k}\,=\, (1/N) \sum _{i} F_{i,i+k}$, where *N* is the number of *F*_*i*,*i*+*k*_ at a given separation *k*. We impose a fixed range on the genomic separation, namely *k*=−*k*_*c*_…*k*_*c*_ with a separation cutoff *k*_*c*_.

The average free energy, $\bar {F}_{k}$ represents the dominant distance dependent energy and results from the free energy cost for making a loop in the DNA with genomic distance, *k*. (Additional distance dependences due to chromatin structure may still remain in *δ* *F*_*i*,*j*_). Polymer physics suggests that $\bar {F}_{k} \sim \alpha \log |k|$ [34], which grows logarithmically with distance. This is akin to the probability of contact as a function of separation for a random polymer going as *p*_*k*_∼|*k*|^−*α*^, with $\bar {F}_{k} \propto -\log {p_{k}}$.

The free energy fluctuations away from the average, *δ* *F*_*i*,*j*_, will contain additive contributions from specific interactions due to chromatin factors, biases due to the protocol and potentially additional distance dependent energies arising from differences in the polymer nature of chromatin at different loci. Principal Component Analysis (PCA) provides a method for decomposing data fluctuations into a linear combination of independent modes. In order to apply PCA, we need a set of observations. Here, the observations correspond to the set of fixed length free energy profiles, one for each bin in the genome. For each bin, *i*, the corresponding free energy profile is the list of interaction energies *F*_*i*,*i*+*k*_ where *k*=−*k*_*c*_…*k*_*c*_ and has a fixed length of 2*k*_*c*_+1. (We also only use those bins *i* that are +/−*k*_*c*_=60 bins from the beginning and end of a chromosome. Thus only a subset of all bins (10819 of the 11546 10 kb bins) are used in creating a list of free energy profiles to be analyzed). Each principal component represents a particular spatial pattern of interaction energy and its corresponding eigenvalue, the amount of variance it accounts for in the free energy fluctuations. We find that the spatial frequency of a given PC increases with decreasing variance. The free energy between bin *i* and *j* can be decomposed using PCA as

(3) $$\begin{array}{@{}rcl@{}}  F_{i,j}&=\bar{F}_{j-i}+ \frac{1}{2} \sum\limits_{\alpha} \left[C^{\alpha}_{i}\phi^{\alpha}_{j-i} + C^{\alpha}_{j} \phi^{\alpha}_{i-j}\right], \end{array} $$

where, $\phi ^{\alpha }_{k}$ is the *α* ^*th*^ eigenvector and only depends on the genomic separation *k*=*j*−*i*. The coefficient, $C^{\alpha }_{i}$, is the projection of the *i* ^*th*^ free energy profile onto the *α* ^*th*^ eigenvector, $C^{\alpha }_{i} = \sum _{k} \phi ^{\alpha }_{k} (F_{i,i+k} - \bar {F}_{k})$. In the analysis that follows we have used a genomic separation cutoff of *k*_*c*_=60 bins which at a resolution of 10 kb corresponds to free energy profiles and eigenvectors that range from [−600,…,600]kb. Matrix elements corresponding to bins *i* and *j* that have |*i*−*j*|>*k*_*c*_ are excluded from analysis. (We have found that for the Drosophila Hi-C data [14] at a resolution of 10 kb, for |*i*−*j*|> 600 kb the statistics of counts becomes too sparse and $\bar {F}$ is not well determined).

We can use PCA to filter out principal components (PCs) that are identifiable with systematic biases or noise, leading to a smoothened set of interaction energies, *δ* *F*. The specific interaction energy can be reconstructed via

(4) $$ \delta F'_{i,j} = \frac{1}{2}\sum \limits_{\beta} \left[C^{\beta}_{i} \phi^{\beta}_{j-i} + C^{\beta}_{j} \phi^{\beta}_{i-j}\right],  $$

where the sum is over only the eigenvectors that are not identified with systematic biases and whose eigenvalues lie above the noise cutoff, and *j*−*i* is restricted to the range [−*k*_*c*_,…,*k*_*c*_].

### Calculating chromatin coupling energies

We model the specific energy of interaction, *δ* *F* *i*,*j*′, between bins *i* and *j* as a sum of pairwise interactions between the bound chromatin factors at those two locations. This can be written as

(5) $$\begin{array}{@{}rcl@{}}  \delta F_{i,j}'&= \sum\limits_{\nu \geq \mu} J_{\mu,\nu} \left[S^{\mu}_{i} S^{\nu}_{j} + S^{\nu}_{i} S^{\mu}_{j}\right], \end{array} $$

where, $0 < S^{\mu }_{i} < 1$ is the occupancy of chromatin factor *μ* at bin *i* (and can be determined from binding data), and *J*_*μ*,*ν*_ is the symmetric coupling energy between chromatin factors *μ* and *ν*.

To obtain the $S^{\mu }_{i}$, we use the locations of enriched regions for a given factor *μ* that are available for download at modencode.org. A given enriched region has a beginning and end genomic coordinate as well as a log-odds score which can be thought of as a binding energy. For a given bin *i* in the genome, the total binding energy $E^{\mu }_{i}$ for factor *μ* is found by adding up the log-odds scores for all of its enriched regions that overlap with the bin. Statistical physics gives a prescription for converting these binding energies into occupancies via $S^{\mu }_{i} = 1/\left (1 + \exp \left [-(E^{\mu }_{i} - \epsilon ^{\mu })/\sigma ^{\mu }\right ]\right)$ where we take *ε* to be the average binding energy over the bound bins, and *σ* the standard deviation which is related to an effective temperature. Given the measured *δ* *F* *i*,*j*′ and $S^{\mu }_{i}$, Eq. 5 presents a linear system that can be fit directly to obtain the *J*_*μ*,*ν*_. We use least-squares fitting to solve this linear system.

## Results and discussion

Using the analysis techniques described in ‘Methods’ we determine the long-range coupling energies, $\bar {J}_{\mu,\nu }$, between a set of chromatin factors by combining the frequencies of interaction as measured by Hi-C and the factors’ genome-wide binding locations. Drosophila makes an excellent model organism on which to test this analysis as the measured Hi-C dataset [14] is of sufficient resolution (down to 10 kb resolution) and there exist a number of measured binding sites for chromatin factors [12,33]. Here we consider insulator associated proteins (BEAF, dCTCF, GAF, Zw5 and CP190) as well as Poly-comb group proteins (Pc, PCL, Pho) that have, respectively, been shown to be responsible for setting up euchromatic [24,25] and heterochromatic [14,26] domains via looping interactions. We also include factors such as Cohesin, Chromator and PolII that are know to be associated with insulators. We will show that our PCA methodology can be used to filter out biases as well as high frequency noise in the Hi-C data. Using our interaction model we assess our PCA normalization procedure against other normalizations methods based on how well it can fit the corrected Hi-C data. In the end a biologically meaningful set of predictions for the effective energetic couplings between chromatin factors is made.

### Distance dependent free energy

One of the challenges in analyzing Hi-C data is the existence of systematic biases due to the measurement protocol and several normalization procedures have been put forward to correct for them. We wish to determine whether these normalization procedures have any effect on the predicted coupling energies between chromatin factors. Using the original published Hi-C dataset for Drosophila [14] we have constructed several different contact matrices at a resolution of 10 kb (see Methods). The contact matrix gives the number of times that a given 10 kb bin is in contact with another, non-overlapping10 kb bin in the genome. We have made a contact matrix based on the original observations, termed *raw* in what follows. In Sexton et al. [14], they presented a hierarchical probabilistic model to correct for various biases in the raw data (see Methods). We have applied this method to the *raw* contact matrix leading to a normalized contact matrix that we label *hierarchical*. We also apply another proposed normalization procedure termed ICE [30] that normalizes each genomic location to have the same total number of observed interactions (see Methods). This leads to three Hi-C contact matrices and they will be labeled as: *raw*, *raw + ICE*, and *hierarchical*. Each of these will be transformed into free energies and filtered by PCA to see if it can improve the fit to the interaction model we presented in Methods and detail below.

**Figure 1:**
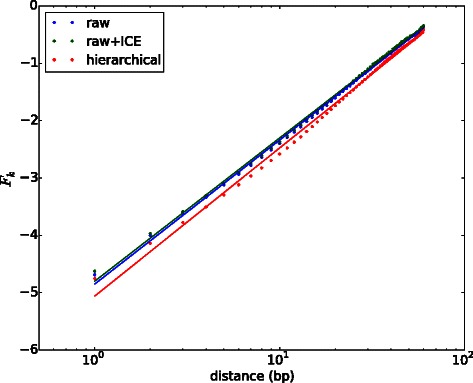
Average free energy of interaction. The genome-wide average free energy, $\bar {F}_{k}$, as a function of genomic separation (a 600 kb window at 10 kb resolution) for free energies derived from three contact matrices (shown in legend). All show that the average free energy cost associated with forming a loop grows with the linear separation between genomic bins. Fitting a polymer model, $\bar {F}_{k}\sim \alpha \log {|k|}$ (see Methods) gives *α*=1.09, 1.085 and 1.12 for the *raw*, *raw + ICE* and *hierarchical* matrices.

For each contact matrix we apply our free energy transformation (see Eq. 1 in ‘Methods’), leading to three different free energy matrices that represent the energetics of interaction between genomic locations. Regardless of whether the contact matrix was normalized or not, the dominant contribution to the free energy is due to the distance dependent entropic cost of looping the DNA polymer between two genomic locations. We determine this distance dependent background free energy, $\bar {F}_{k}$ by averaging together all free energy matrix elements *F*_*i*,*j*_ that are at a fixed genomic separation *k*=*j*−*i* (see Methods). In Figure 1 we plot $\bar {F}_{k}$ for the three different free energy matrices used in the analysis. It can be seen that the free energy associated with this looping increases with the linear separation. We have fit each of the three average free energies to the prediction for that of a random polymer, namely that $\bar {F}_{k} \sim \alpha \log |k|$, where *α* is the scaling exponent. For an ideal random polymer in 3D, the scaling exponent would be predicted to be *α*=3/2. From the Drosophila Hi-C data, we find that the four matrices have average free energies that have roughly the same scaling (*α*=1.1±0.1). This result is in agreement with that found for other Hi-C datasets where, *α*∼1. We now show how the free energy fluctuations around the average can be further decomposed into an independent set of interaction modes using Principal Component Analysis (PCA).

### PCA of free energy profiles

From each free energy matrix, we create a list of free energy profiles, one for each genomic bin. A given free energy profile shows how that particular genomic segment interacts with the surrounding region (seeMethods). Besides the background free energy above, each profile will have free energy fluctuations, *δ* *F*, that are potentially due to interactions between bound factors, or systematic biases. We use PCA to identify the independent contributions to the fluctuations in the free energy. The top principal components (PCs) represent common patterns of interaction that are present at many locations in the genome. The aim is to then identify which PCs represent systematic effects as well as those that are just associated with noise in the Hi-C data. These can then be filtered out to create a corrected set of free energy profiles.

**Figure 2:**
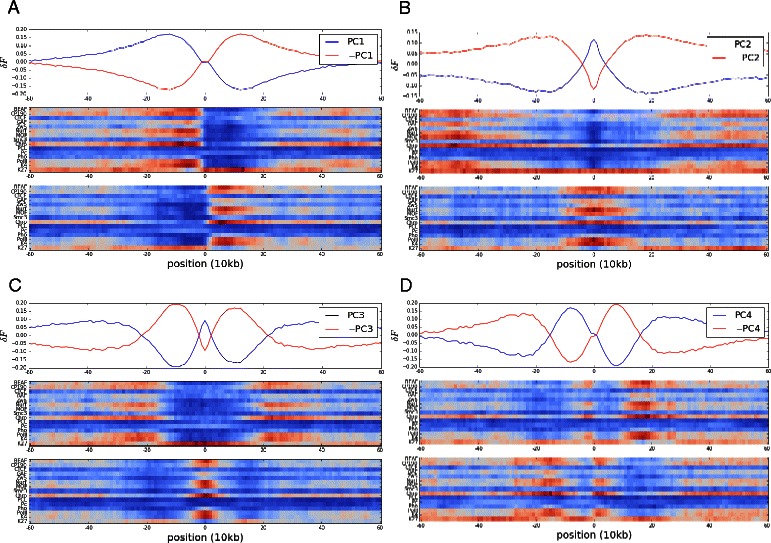
Free energy principal components and chromatin-binding profiles. Shown are the first four principal components **(A, B, C, D)** calculated genome-wide from the *F*_*i*,*j*_ matrix created from the *raw + ICE* contact matrix (top plots). Below each free energy profile are heat maps of the genome-wide average binding profiles for the selected chromatin factors (see Text). The top heat map corresponds to the positive free energy interaction profile (blue curve), and the bottom heat map for that of the inverse profile (red curve). Red regions in the heat maps represent locations of higher occupancy and blue regions represent lower occupancy. The range of the heat maps goes from 0.0 (blue) to 1.0 (red).

We performed PCA on each of the three matrices. In Figure 2 we show the top four principal components (PCs) for the *raw + ICE* free energy matrix. Each PC shows the variation in the free energy as a function of the genomic separation from the bin located at *k*=0. Positive free energies correspond to repulsive interactions whereas negative ones are attractive, and thus represent stabilizing interactions. It should also be noted that for each PC there is also the inverse interaction profile that is obtained by multiplying the PC by −1. These PCs can also be interpreted as a set of spatial modes with which to represent the data, akin to a Fourier decomposition. The characteristic spatial frequency of a PC increases as the corresponding eigenvalue (variance) associated with it decreases. Many of the PCs corresponding to small eigenvalues represent high-frequency noise. In what follows, we show that this noise can be filtered out by reconstructing the specific interaction energies (Eq. 4) without including them in the sum. The PCs resulting from the different free energy matrices are similar but do have key differences as shown in Additional file 1: Figure S2. (We note that the top PCs still emerged if a smaller subsample of free energy profiles was used, reducing the effect of nearby correlated bins, see Additional file 2: Figure S3). For example, if ICE normalization has not been performed on the *raw* matrix the first PC is an overall constant offset since the bins of the free energy matrix have different means. Also the spatial frequencies differed between the PCs derived from the *raw* or *raw + ICE* matrices compared to those from the *hierarchical* matrix. We attribute this to the distance scaling correction that is applied in the hierarchical normalization method. This will turn out to have consequences in how well the interaction model fits the hierarchical normalized Hi-C data.

### Interaction profiles and chromatin structure

Prior PCA analysis on Hi-C data highlighted the existence of chromatin compartments, namely topological domains that have interactions amongst themselves but not with each other [13]. The insulator and polycomb group factors of interest in this manuscript are thought to interact to generate such compartmentalization. These domains, which are strongly associated with euchromatin or heterochromatin, can exist on a range of scales. Our free energy PCs also show such a structure, with the first (or second, depending on normalization) PC marking a domain between such compartments. With increasing number, and decreasing variance in free energy, the PCs show such interactions at smaller and smaller spatial scales. As such, PCs form a basis with which to decompose a given energy interaction profile into various spatial scales.

In order to help clarify the interpretation of the energy interaction profiles represented by each PC, we look at the distribution of bound chromatin factors associated with each PC. In Figure 2, we show the PCs calculated from the *raw + ICE* free energy matrix and the corresponding average binding profiles of our selected chromatin factors as heat maps. Each average binding profile was computed using only those genomic locations where the specific energy interaction profile had a significant projection onto the given PC. In particular, the energy profile can have either a negative or positive projection (see Additional file 3: Figure S1, B), and so we create two sets of bins: those bins, *i*, that have projections $C^{\alpha }_{i} > 2 \sigma _{\alpha }$ and those with projections $ C^{\alpha }_{i} < - 2\sigma _{\alpha }$ (corresponding to the inverse PC profile), where $\sigma _{\alpha }^{2}$ is the eigenvalue (variance) of the *α* ^*th*^ PC. We then extract binding profiles ($S^{\mu }_{i}$) from the genome-wide binding data of each chromatin factor that are centered on each set of locations. These then get averaged together to give the average binding profile that shows the underlying chromatin structure associated with the given principal component computed from the Hi-C data.

For example, for the *raw + ICE* free energy matrix, PC1 represents a domain boundary between euchromatin (marked by H3K4me3) and heterochromatin (marked by H3K27me3). Those genomic locations that have a positive projection onto PC1 (middle heat map) have euchromatin factors bound on the left and heterochromatin factors bound on the right. Looking at the associated free energy, euchromatic DNA shows a larger cost in free energy (positive values) associated with looping likely due to it having greater entropy, due to being more open and hence more disordered. As such, PC1 may represent the mutual exclusion of interactions between euchromatin and heterochromatin domains that are physically insulated from one another [13,14]. Figure 2 shows that for the top PCs derived from the *raw + ICE* matrix, strong correspondences exist between the type of the interaction and the underlying bound factors (i.e. locations that are bound by insulator factors have attractive (negative) interactions with other domains bound by insulators). We found similar strong statistics (see Additional file 4: Figure S4) for the PCs derived from the *raw* free energy matrix, but found much weaker correlations between the PCs and underlying bound factors for the *hierarchical* matrix.

### Coupling energies between chromatin factors

Using PCA we can filter out various PCs (using Eq. 4), yielding corrected specific interaction energies *δ* *F* *i*,*j*′ between locations *i* and *j* (see Methods). For matrices not treated with ICE, the first PC simply represents a DC offset that is present in each energy profile. This corresponds to the biases identified by ICE. In reconstructing the specific interaction energies, *d* *F* *i*,*j*′, we leave out this PC for the non-ICEd matrices. Reconstructing the interaction energies using a subset of the remaining PCs, smooths the data and filters out noise. We now assess how much filtering to perform based on how well the pairwise interaction model fits the data.

**Figure 3:**
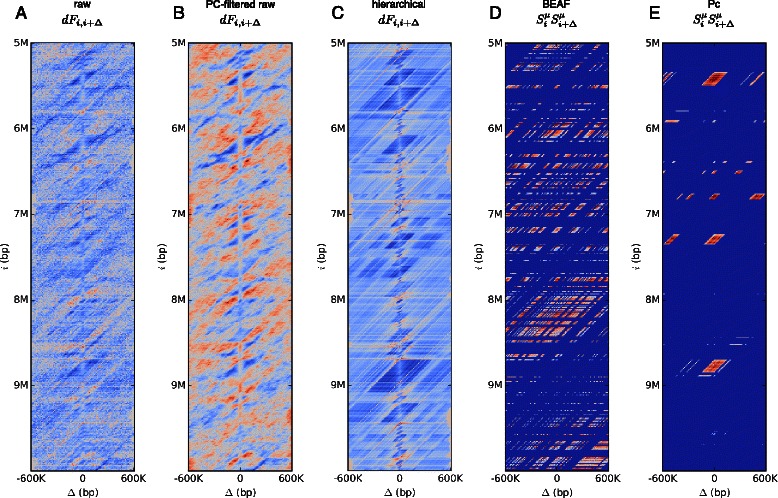
Specific energies of interaction and associated chromatin factor contacts. Shown in **(A, B, C)** are the energies of interaction $\delta F_{i,j} = F_{i,j} - \bar {F}_{j-i}$ of a portion of chromosome 2L for three different *F*_*i*,*j*_ matrices: A) *raw*, B) *raw + PC* filtered and *hierarchical*. (The first 35 PCs were used in reconstructing the *raw + PC* free energies). All have been aligned so that the zeroth column corresponds to *i*=*j*. Blue regions correspond to attractive interactions (negative) and red regions to effective repulsive interactions (positive). Figures **(D, E)** show the locations of pairwise self contacts, $S_{i}^{\mu } S_{j}^{\mu }$ for the insulator factor BEAF and the polycomb group protein Pc (blue corresponds to $S_{i}^{\mu } S_{j}^{\mu } = 0$ and red to $S_{i}^{\mu } S_{j}^{\mu } = 1$). Comparing the interaction energies **(A, B, C)** with the locations of pairwise contacts **(D, E)** highlights how these contacts could be generating the observed interactions.

In Figure 3(A-C) we show the specific interaction energies for a portion of chromosome 2L. As can be seen PCA filtering dramatically smoothens the data, highlighting domains of attractive (blue) and repulsive interactions (red). As a comparison we show the energies computed from hierarchical normalized data for the same region. The two normalized energy matrices agree in many domains, but do possess differences, such as the size of the interacting domain situated around 9 Mb. Many of these interactions are due to specific contacts between chromatin factors at the given loci. We highlight this connection by showing the pairwise self contacts, $S_{i}^{\mu } S_{j}^{\mu }$, for the same region for the insulator BEAF and the polycomb factor Pc (Figure 3D,E). For example, some of the attractive energies (blue region near 8Mb in the *δ* *F*_*i*,*j*_ heat maps) are likely due to interactions between insulators (BEAF-BEAF domain in Figure 3D), whereas other attractive interactions (region between 5 Mb and 6 Mb) could be due to interactions between the polycomb group of factors (Pc-Pc domain in Figure 3E). We now assess how well the interaction energies are fit to a model that takes the distribution of contacts between bound factors as input.

**Figure 4:**
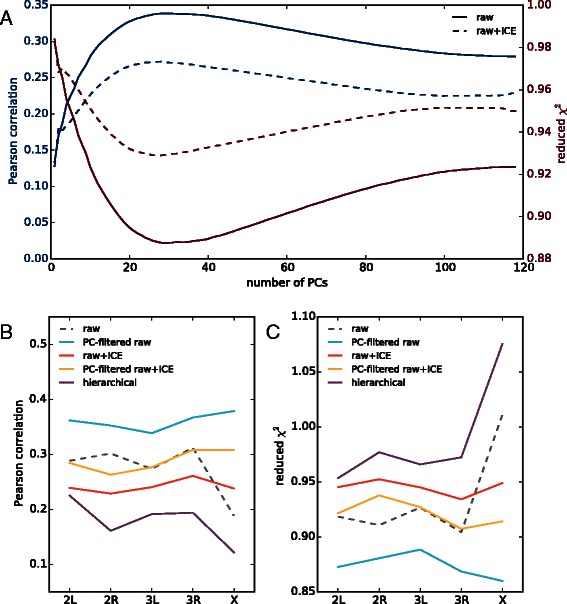
PCA filtering can improve fit to interaction model.**A)** There is an optimal number of PCs to use in reconstructing the energies of interaction *δ* *F*_*i*,*j*_. Shown are the Pearson correlation coefficient and reduced *χ* ^2^ of the genome-wide fits of the given data (see legend) to the model using Eq. 5 as a function of the number of PCs used in the filtering. For the energies derived from *raw* matrix, PC1 was excluded as it is simply a DC offset. **(B, C)** show the best fit results for the various datasets by chromosome. PCA filtering for the *raw* matrix leads to the best overall results.

For each set of interactions energies, either filtered by PCA or some other normalization method, we fit Eq. 5 to determine a fitted set of coupling energies *J*_*μ*,*ν*_. (We have fit all the chromosomes at once, as well as chromosome by chromosome, allowing us to determine how much the fitted *J*’s vary by chromosome). We use *χ* ^2^ and the Pearson correlation coefficient to determine how much PC filtering, if any should be applied to the interaction energies. (All of the fits are statistically significant, as determined by a permutation test, which gave *r*∼0). In Figure 4, we show that for the interaction energies derived from the *raw* and *raw + ICE* matrices, that PCA filtering can improve the quality of the fit. Figure 4A shows that using the first 35 PCs leads to the best genome-wide fit of the data by the model (for the non-ICED matrix, we also left out the DC offset PC). Interestingly, using ICE reduced the overall quality of the fit compared to the *raw* matrix, though PC filtering was able to improve the fitting for both. This reduction in fit quality is potentially not surprising as any normalization method is removing information present in the original data. We found that applying any form of PC filtering to the interaction energies derived from the hierarchical normalized matrix always made the fit worse. As a summary, in Figure 4B,C we show the chromosome by chromosome *χ* ^2^ and Pearson correlation coefficient for the various fits of the model to both PC filtered and unfiltered data. PC filtering of the energies computed from the *raw* matrix give the best overall fit. The distance dependent scalings applied in the hierarchical normalization method lower the correlation between the interaction energies and the underlying bound chromatin factors, lessening the quality of the fit.

**Figure 5:**
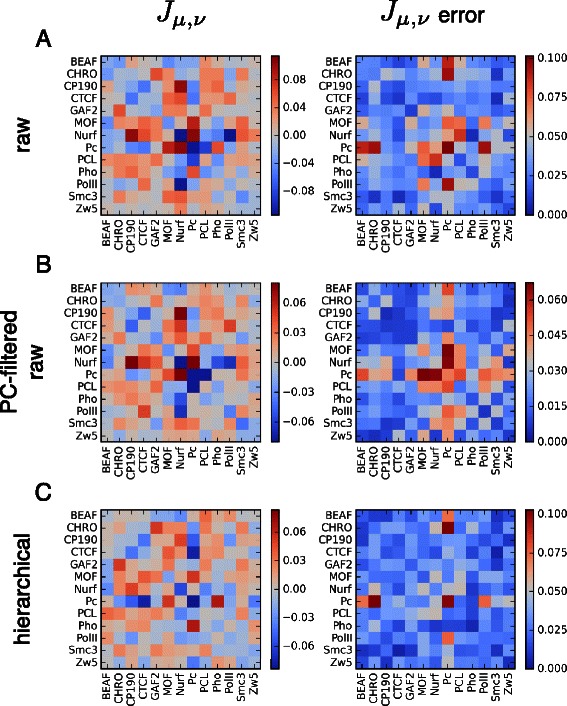
Chromatin factor coupling energies from fitting. The fitted coupling energies, *J*_*μ*,*ν*_, between chromosome associated factors. The left heat maps show the chromosomal average *J*’s, and the right heat map the associated standard deviations in the average values. The following free energy matrices were used: **A)** *raw*, **B)** *raw + PC* filtering (optimal number of PCs used was 35) and **C)** *hierarchical*.

In Figure 5, we show the fitted coupling energies from the fits to the *raw*, *raw + PC* filtered and *hierarchical* data. As mentioned PCA filtering improved the fit, yet the resulting *J*’s show an overall agreement between the different data sets. Here we show the average *J*’s over all the chromosomes (left heat maps) and their associated standard deviations (right heat maps). The parameter error estimates show that many of the couplings are consistently predicted from one chromosome to the next. An inspection of the fitted couplings that are consistent across chromosomes show that many of the insulators and factors that are linked to euchromatic domains have attractive (negative) interactions, speaking to their ability to stabilize loops in such domains [24,25]. Many of these have effective repulsive (loop hindering) interactions with polycomb group proteins (PCL, Pc), though some have attractive interactions with Pho. Other things that are shared between these sets of *J* are the associations between BEAF, Chromator and Cohesin and the transcriptional machinery factors, PolII and Nurf. Interestingly, the predicted interactions between CTCF and such factors are more complex, highlighted by the effective positive interactions. We should also point out that a given *J* represents a pair’s effect on looping and should not be interpreted as a prediction of whether they interact or not. Factors may very well interact (i.e. have attractive protein-protein interactions) but yet have a destabilizing effect on loop formation. We note that within both the insulator and polycomb group, some pairs of factors are predicted to effectively raise the energy of loop formation. We also point out that other models could also be fit, for instance leaving out self-interactions, that may help to reveal more specific interactions, though potentially reducing the quality of the fit.

It should be recalled that these are interactions determined at a resolution of 10 kb, so factors that might juxtapose side-by-side at boundaries that form on finer length scales would get grouped together. Experiments that probe at finer resolutions would be valuable in sorting out potential conglomerated interactions. Nevertheless, our findings highlight how using PCA can help improve the quality of fit of Hi-C data to a model for chromatin factor interactions and that a consistent set of couplings can be predicted, which can be explored experimentally.

## Conclusions

In this paper we have described a method for normalizing Hi-C data using principal component analysis (PCA). PCA decomposes the free energy into various contributions, including a distance dependent entropic free energy, potential systematic biases, and specific energies of interaction potentially arising from DNA bound factors. We assessed the performance of the PCA based normalization method, along with two others, by fitting the corrected data to a pairwise interaction model that took as input the locations of bound chromatin factors. This allowed us to determine the coupling energies between chromatin bound factors from the energies of interaction as determined from Hi-C data. As a test case, we calculated the couplings between insulators, polycomb-group, other chromatin factors and some of the transcriptional machinery. These factors are responsible for setting up the domains/compartments in the DNA, yielding the two compartmental model that can broadly classify chromatin structure. Recent work has shown that a simple A/B interacting copolymer model can capture many of the observed patterns found in Hi-C data [35]. Polymer simulations including a simple insulator interaction has also shown how compartments can be formed [36]. Our work, is a first step toward trying to break apart the interactions within such compartments into their constitutive parts. The couplings found here could help further such simulations by including a richer set of interactions. Of course, this requires a reliable set of predictions for interactions and we have shown that correcting the Hi-C data using PCA, can improve the quality of the fit.

The methods presented here are readily applicable to the Hi-C and bound factor data obtained in other organisms, and should provide a common framework in aiding the correction and ultimate functional analysis of such data. Our work may thus provide the community with a valuable tool not only to predict the strength of Hi-C interactions due to chromatin associated factors, but also to better evaluate the specific variations encountered depending on cellular contexts and/or conditions.

## Additional files

Additional file 1
**Figure S2.** Principal component analysis on free energy matrices. First five principal components derived from *δ* *F*_*i*,*j*_ matrix. **(A)**, **(B)**, **(C)** and **(D)** respectively represent the data from the contact matrices *raw + ICE*, *raw*, *hierarchical + ICE* and *hierarchial* (see Methods).

Additional file 2
**Figure S3.** Sub-sampling yields similar PCs. First nine principal components derived from the free energy profiles created from the *raw* matrix. Shown in red are the PCs calculated from sub-sampling a 1/3 (= 3500) of the free energy profiles.

Additional file 3
**Figure S1.** PCA spectrum and projections. **(A)** Spectrum of eigenvalues (variances) for the *raw + ICE* free energy matrix. **(B)** Histogram of projection of bins in *δ* *F*_*i*,*j*_ on the first PC. (Left inset) Free energy profile for a genomic location with a large negative projection and (right inset) a location with large positive projection.

Additional file 4
**Figure S4.** Statistics between bound factors and free energy principal components. Statistics on the correlation between the PC’s derived from the *raw + ICE* matrix with the selected chromatin factors. For each binding factor and each PC, the Kolmogorov-Smirnov test, was performed to test the projections for all bins bound by that factor to those that were not. The first column is the P-value, and the next column is the percentage of bins bound by the factor with projections >2.*σ* and the last column represents fraction of bins bound by the factor with projections <−2.*σ* projection.
