# Supplementary figures and images for: Probing long-range interactions by extracting free energies from genome-wide chromosome conformation capture data
Source: BMC Bioinformatics. 2015 May 23;16:171. doi: 10.1186/s12859-015-0584-2 (PMC4492175; doi:10.1186/s12859-015-0584-2)

(A)

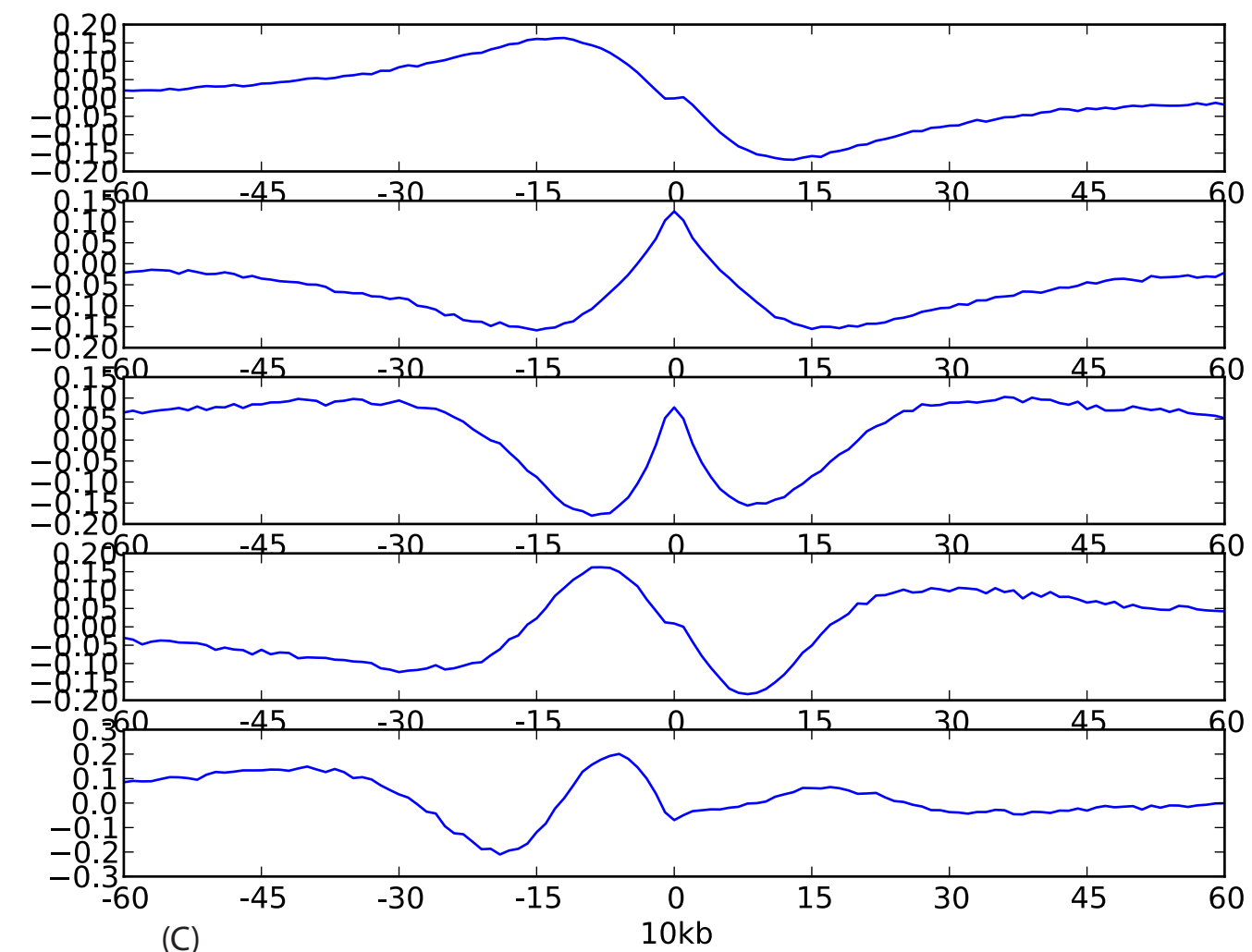

(B)

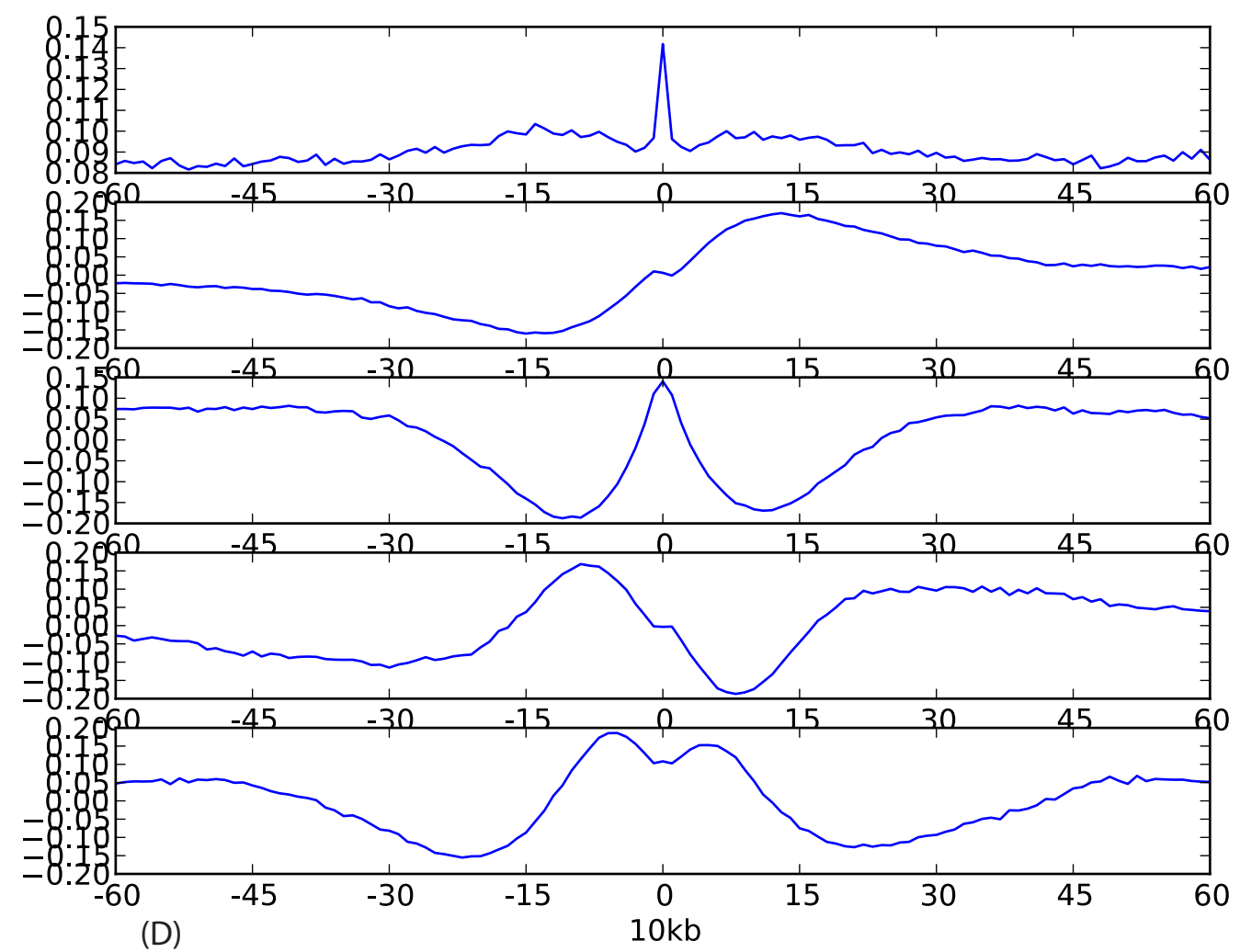

(C)

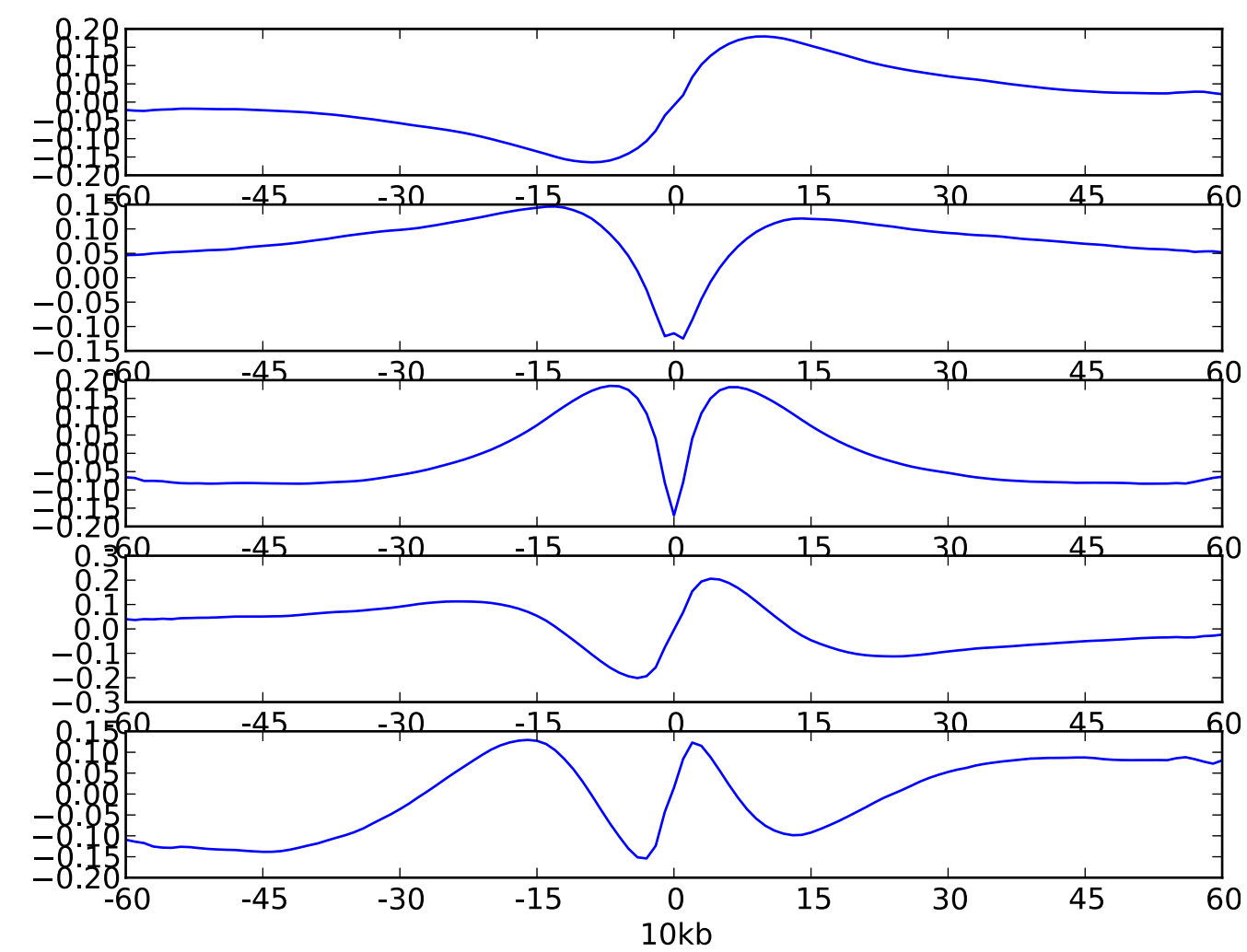

(D)

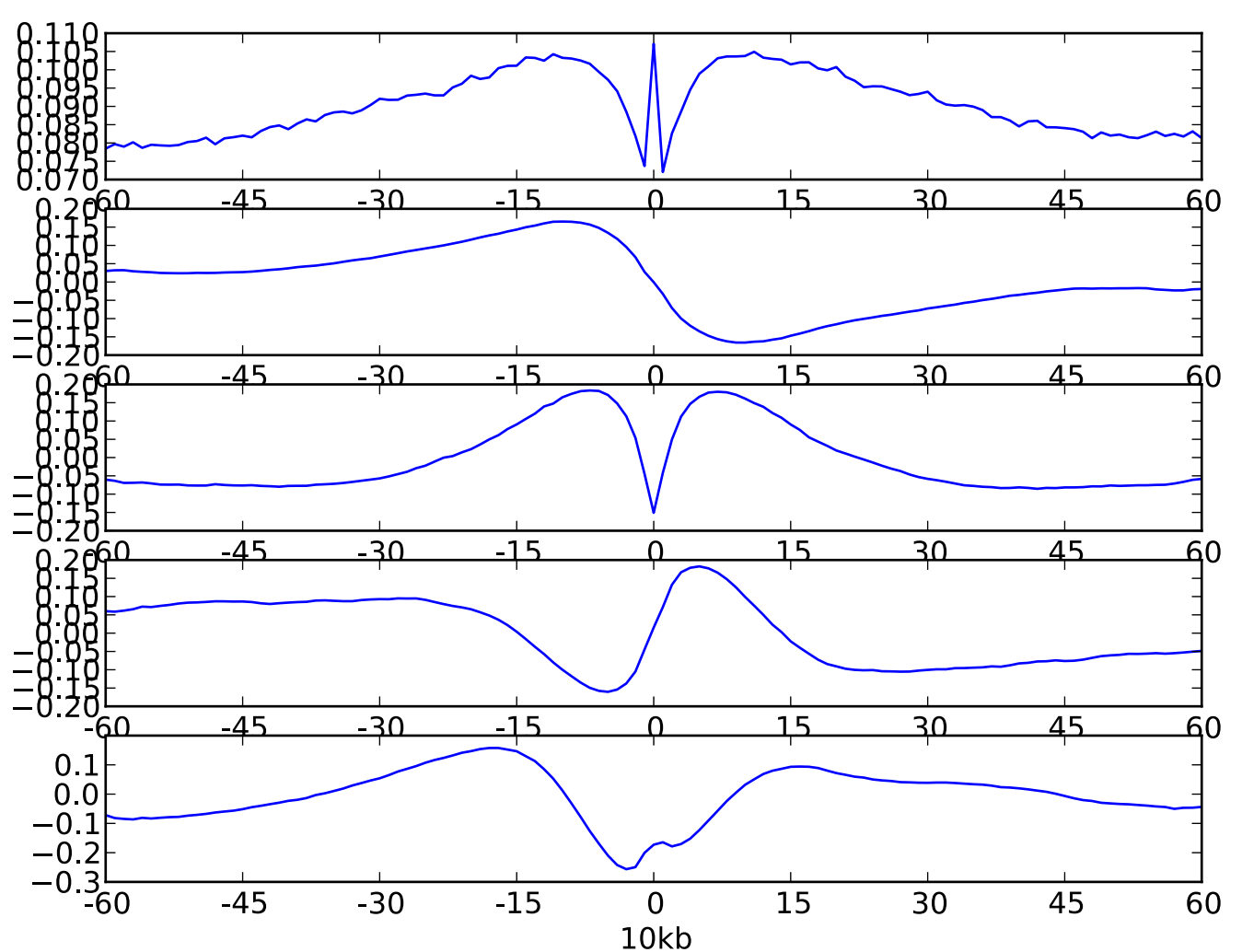

Supplement: Additional file 1 — Figure S2. Principal component analysis on free energy matrices. First five principal components derived from δ F i,j matrix. (A), (B), (C) and (D) respectively represent the data from the contact matrices raw + ICE, raw, hierarchical + ICE and hierarchial (see Methods). [file 12859_2015_584_MOESM1_ESM.pdf]

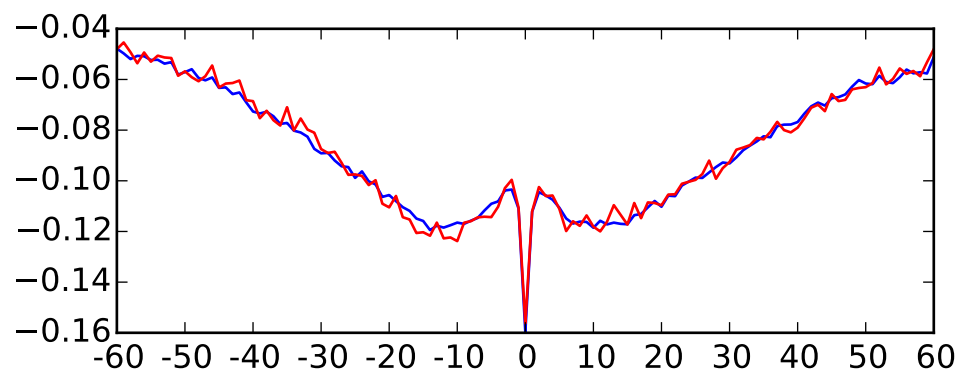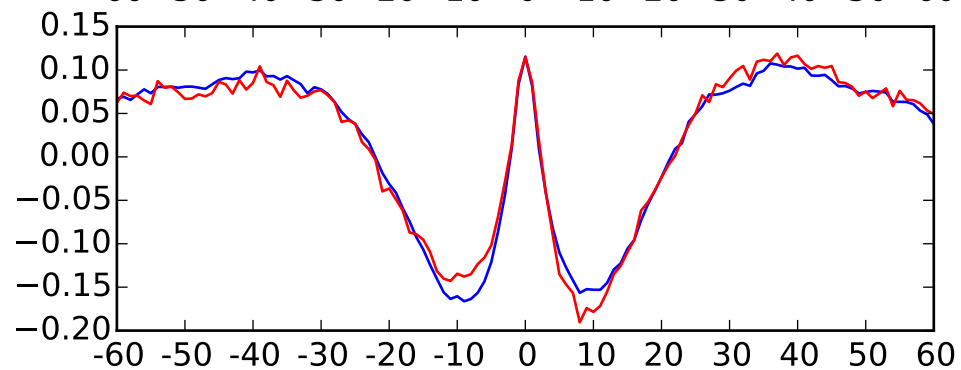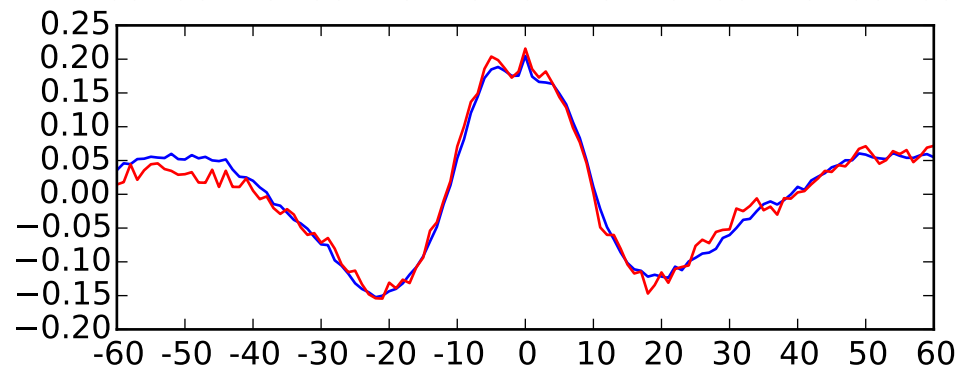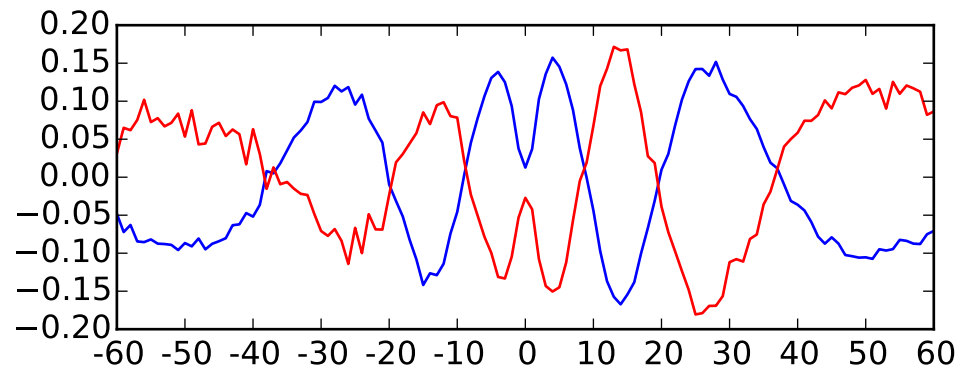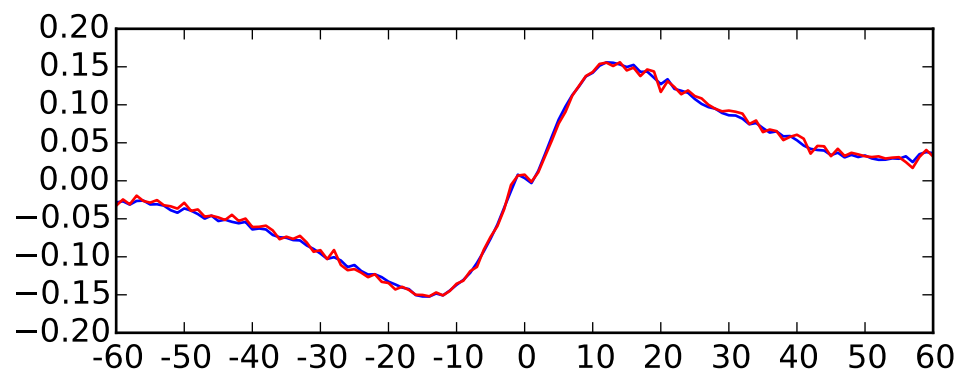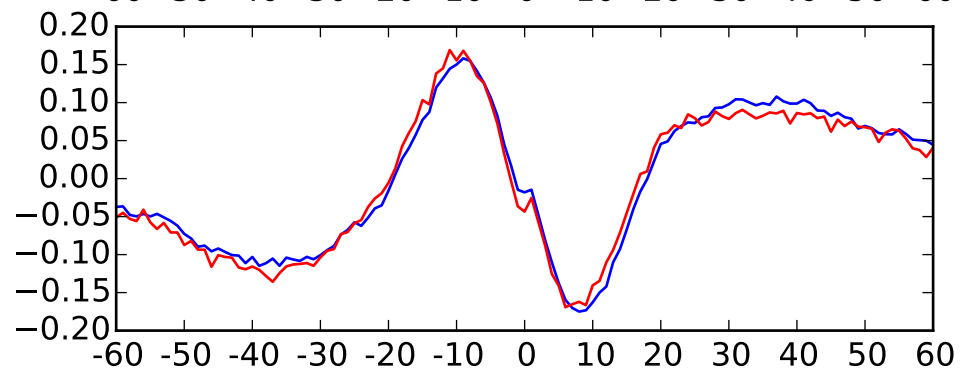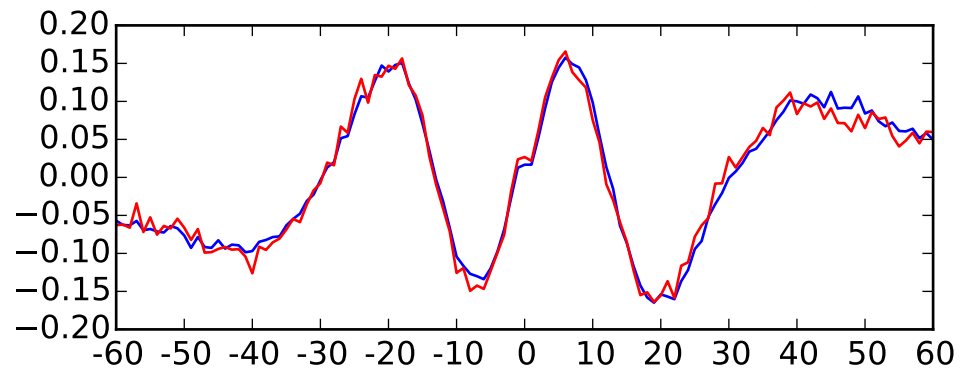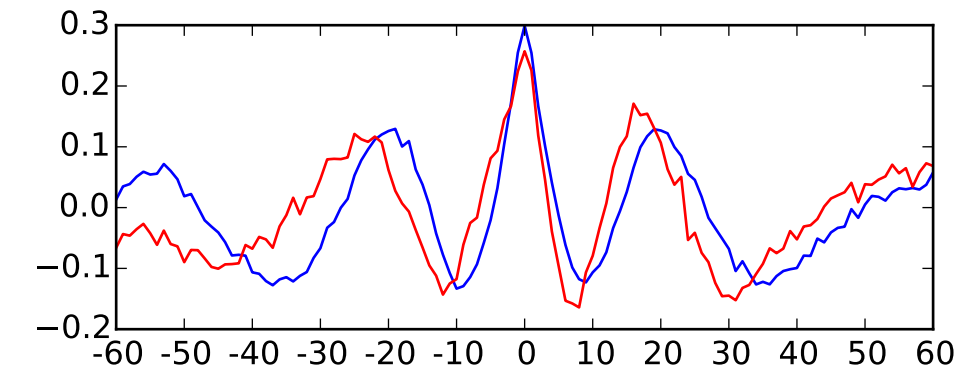

Supplement: Additional file 2 — Figure S3. Sub-sampling yields similar PCs. First nine principal components derived from the free energy profiles created from the raw matrix. Shown in red are the PCs calculated from sub-sampling a 1/3 (= 3500) of the free energy profiles. [file 12859_2015_584_MOESM2_ESM.pdf]

(A)

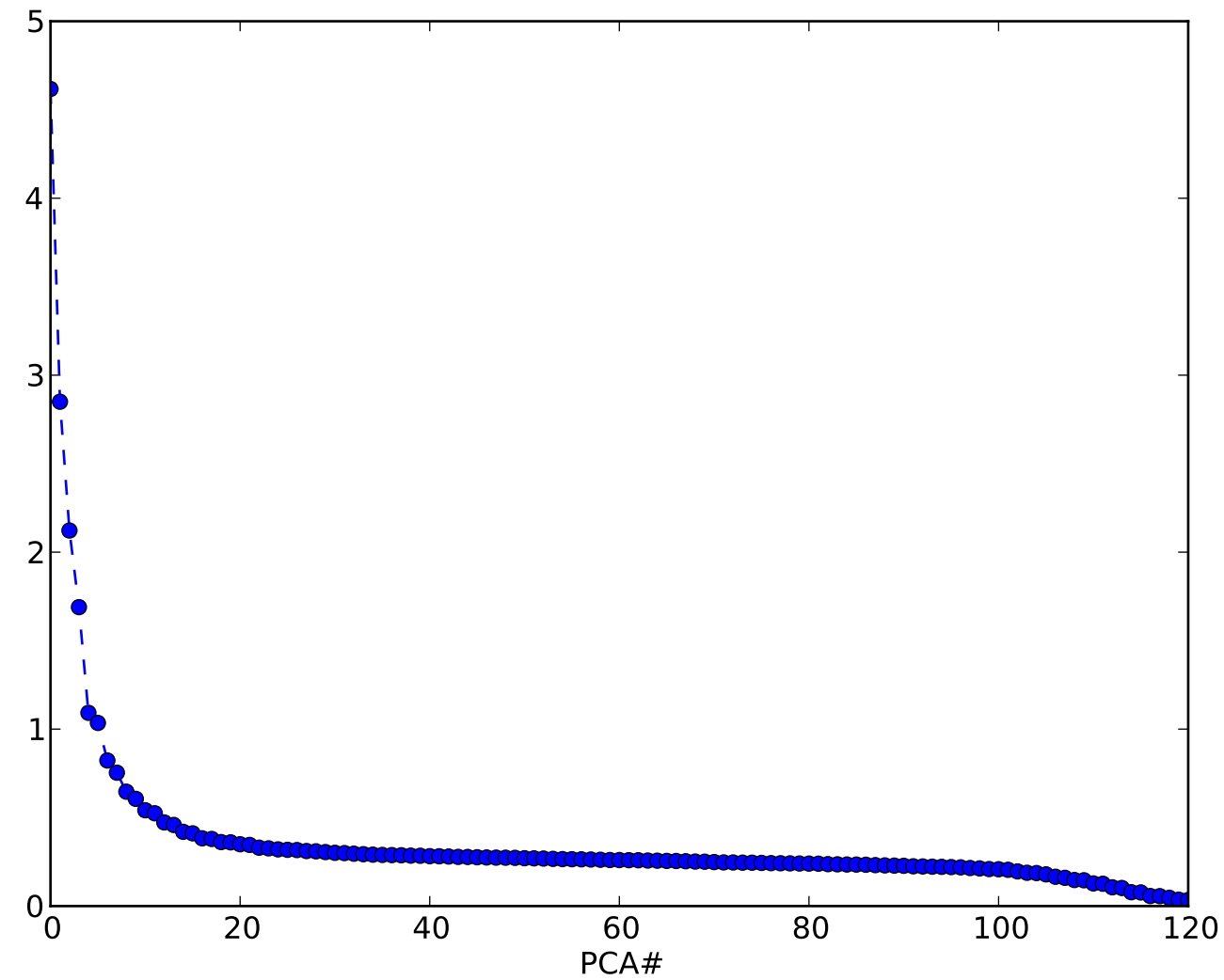

(B)

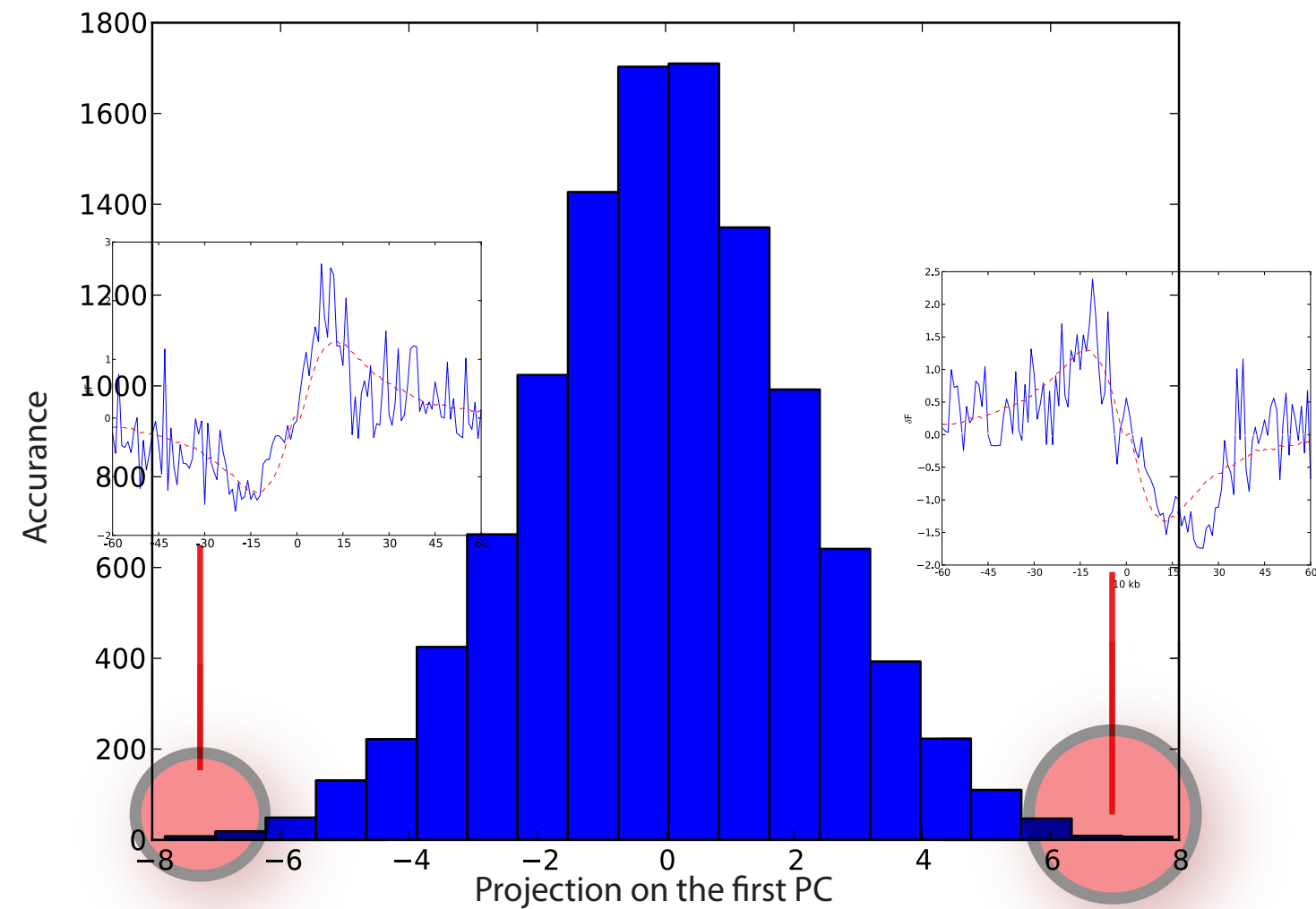

Supplement: Additional file 3 — Figure S1. PCA spectrum and projections. (A) Spectrum of eigenvalues (variances) for the raw + ICE free energy matrix. (B) Histogram of projection of bins in δ F i,j on the first PC. (Left inset) Free energy profile for a genomic location with a large negative projection and (right inset) a location with large positive projection. [file 12859_2015_584_MOESM3_ESM.pdf]
