# Supplementary material for: Probing long-range interactions by extracting free energies from genome-wide chromosome conformation capture data
Source: BMC Bioinformatics. 2015 May 23;16:171. doi: 10.1186/s12859-015-0584-2 (PMC4492175; doi:10.1186/s12859-015-0584-2)
Supplement: Additional file 4 — Figure S4. Statistics between bound factors and free energy principal components. Statistics on the correlation between the PC’s derived from the raw + ICE matrix with the selected chromatin factors. For each binding factor and each PC, the Kolmogorov-Smirnov test, was performed to test the projections for all bins bound by that factor to those that were not. The first column is the P-value, and the next column is the percentage of bins bound by the factor with projections >2.σ and the last column represents fraction of bins bound by the factor with projections <−2.σ projection. [file 12859_2015_584_MOESM4_ESM.pdf]

pc#\BEAF70	P-value	#of rows>2σ bound	#of rows<2σ bound
1	5.52E-08	1.44%	0.72%
2	5.66E-48	0.33%	16.07%
3	7.04E-28	2.92%	20.36%
4	0.85985	9.34%	8.06%
5	2.62E-09	16.94%	4.04%
6	1.71E-05	14.98%	5.69%
7	4.09E-21	2.83%	25.36%

pc#\H3K9me	P-value	#of rows>2σ bound	#of rows<2σ bound
1	0.012602	36.33%	40.94%
2	3.16E-07	50.00%	43%
3	0.413788	34.16%	34.91%
4	0.506762	42.80%	41.13%
5	2.87E-07	33.88%	47.79%
6	0.000685	33.04%	42.81%
7	0.671483	34.41%	39.64%

pc#\CP190	P-value	#of rows>2σ bound	# of pc<2σ bound
1	1.84E-08	15.47%	21.74%
2	2.17E-39	12.25%	39.73%
3	3.28E-30	14.58%	47.27%
4	0.217097	24.12%	27.42%
5	1.04E-08	33.06%	19.85%
6	1.48E-07	43.61%	23.41%
7	5.00E-19	16.60%	41.43%

pc#\H4K16ac	P-value	#of rows>2σ bound	# of pc<2σ bound
1	1.74E-05	54.68%	62.32%
2	1.57E-07	58.28%	66.96%
3	3.14E-15	49.17%	74.91%
4	0.056105	58.75%	62.10%
5	0.161984	57.44%	65.07%
6	0.121174	63.44%	67.56%
7	8.93E-14	52.23%	75.00%

pc#\CTCF	P-value	#of rows>2σ bound	# of pc<2σ bound
1	0.394739	15.11%	12.68%
2	0.194057	14.90%	8.48%
3	0.520865	11.67%	16.36%
4	0.303585	11.67 % 1	53.31%
5	0.082068	13.64%	90.93%
6	0.390867	12.33%	11.71%
7	0.029746	14.98%	15.00%

pc#\MOF	P-value	#of rows>2σ bound	# of pc<2σ bound
1	3.98E-16	7.55%	12.68%
2	2.25E-14	21.32%	53.57%
3	5.19E-59	12.50%	53%
4	8.43E-05	21.79%	25.00%
5	4.24E-29	43.39%	12.50%
6	1.07E-23	46.70%	16.05%
7	7.04E-32	13.36%	50.36%

pc#\GAF	P-value	#of rows>2σ bound	# of pc<2σ bound
1	8.25E-11	11.51%	14.13%
2	2.17E-81	2.65%	37.50%
3	1.57E-20	19.17%	31.27%
4	8.14E-06	14.40%	18.55%
5	5.79E-13	27.27%	8.82%
6	1.07E-12	31.28%	11.71%
7	3.48E-13	10.93%	27.86%

pc#\SMC3	P-value	#of rows>2σ bound	# of pc<2σ bound
1	5.58E-06	5.40%	4.71%
2	3.81E-57	0.00%	29%
3	2.22E-08	10.83%	20.36%
4	8.10E-05	8.56%	8.87%
5	1.50E-13	11.57%	3.31%
6	4.94E-11	17.18%	6.69%
7	1.19E-10	4.86%	17.50%

pc#\H3K27me3	P-value	#of rows>2σ bound	# of pc<2σ bound
1	7.37E-07	61.87%	59.42%
2	6.55E-92	37.09%	88.84%
3	0.000193	68.75%	76.00%
4	0.013087	61.09%	63.71%
5	5.91E-06	71.90%	63.60%
6	0.010528	69.60%	64.55%
7	9.39E-10	61.13%	77.50%

pc#\H3K4me3	P-value	#of rows>2σ bound	# of pc<2σ bound
1	3.63E-08	33.81%	32.97%
2	1.93E-37	37.09%	57.14%
3	1.98E-48	26.67%	65.82%
4	0.061016	42.02%	42.74%
5	0.007387	48.35%	44.12%
6	0.015079	55.07%	46.82%
7	1.17E-25	34.82%	66.79%
